# Supplementary figures and images for: Prognostics for pain in osteoarthritis: Do clinical measures predict pain after total joint replacement?
Source: PLoS One. 2020 Jan 8;15(1):e0222370. doi: 10.1371/journal.pone.0222370 (PMC6948829; doi:10.1371/journal.pone.0222370)

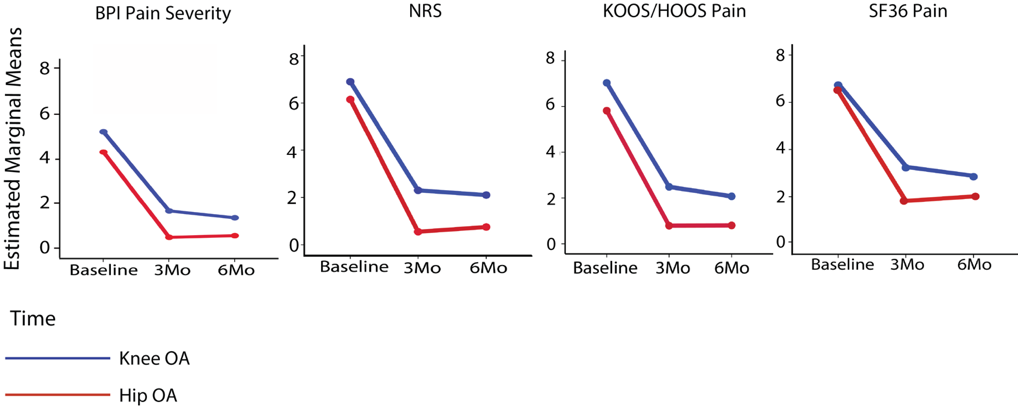

Supplement: S1 Fig — Interaction between the 2 OA groups, time (baseline, 3 months and 6 months), and measurement type (information present in Table 3). HOA patients presented larger pain relief than KOA. For both groups, pain intensity ratings did not show meaningful differences between 3- and 6-months post-surgery. Mo, months. (TIF) [file pone.0222370.s001.tif]

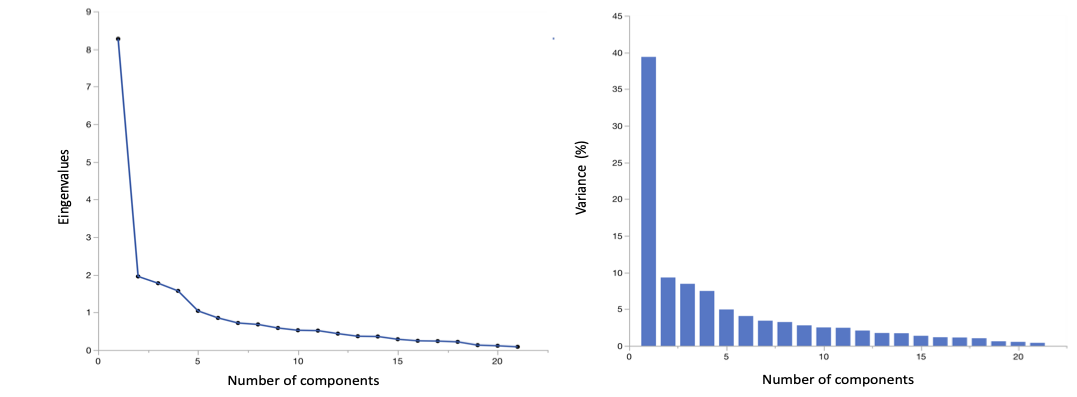

Supplement: S2 Fig — a. Scree plot and percentage of variance explained by each component. The eigenvalues become lower than 1.0 at the fifth component, and the slope of eigenvalues flattens at this component. Components were retained only for eigenvalues higher than 1, corresponding to a percentage of variance higher than 5%. A total variance of 69.54% was explained by the 5 selected components (TIF) [file pone.0222370.s002.tif]
