# Supplementary material for: Prognostics for pain in osteoarthritis: Do clinical measures predict pain after total joint replacement?
Source: PLoS One. 2020 Jan 8;15(1):e0222370. doi: 10.1371/journal.pone.0222370 (PMC6948829; doi:10.1371/journal.pone.0222370)
Supplement: S1 Table — Threshold of factor loading was set on 0.5/-0.5 after Promax oblique rotation (bold). 6MWT = 6 minute walking test; DN4 = The Neuropathic Pain 4 questions; HADS(A) = The Hospital Anxiety and Depression Scale, Anxiety; HADS(D) = The Hospital Anxiety and Depression Scale, Depression; KOOS = Knee Injury and Osteoarthritis Outcome Score, (ADL–Function in daily living), (S -Knee Symptoms), (SR—Function in sport and recreation), (QOL—knee related quality of life); MPQ = McGill Pain Questionnaire, (A–Affective score) (S–Sensory score); PCS = Pain Catastrophizing Scale, (R–Rumination subscale), (M–Magnification subscale), (H–Helplessness subscale); SF36 = Short-form (36) Health Survey, (PF–Physical Functioning), (PH–physical role functioning), (EP–emotional role functioning), (EF–energy/fatigue), (E–emotional well-being), (SF–social functioning), (GH–general health); TUG = Test stand-up and go. (DOCX) [file pone.0222370.s003.docx]

|  | | **Factor 1** | **Factor 2** | | **Factor 3** | **Factor 4** | | **Factor 5** |
| --- | --- | --- | --- | --- | --- | --- | --- | --- |
|  | | **Health** | **Pain**  **Quality** | | **Pain Catastrophism** | **Physical Performance** | | **Affect** |
| **HADS(A)** | 0.1716056 | | 0.1662774 | 0.0878845 | | | 0.0725140 | **0.7154581** |
| **HADS(D)** | 0.2974458 | | 0.0646839 | 0.0542344 | | | 0.1274329 | **0.5458700** |
| **DN4** | -0.0216157 | | **0.6065968** | 0.0700990 | | | -0.1300625 | 0.1298646 |
| **MPQ(S)** | -0.2259922 | | **0.7435562** | 0.0818952 | | | -0.1135575 | 0.0112932 |
| **MPQ(A)** | 0.0929373 | | 0.3093876 | 0.1553277 | | | -0.1234311 | 0.0713686 |
| **KOOS(S)** | 0.1247400 | | **0.6513952** | -0.0126017 | | | 0.1103185 | 0.0985363 |
| **KOOS(ADL)** | 0.1706909 | | 0.3895259 | -0.0740881 | | | 0.1685821 | 0.0088291 |
| **KOOS(SR)** | 0.0884324 | | **0.6775176** | 0.0965312 | | | -0.0413376 | -0.0768334 |
| **KOOS(QOL)** | 0.1352021 | | **0.6553364** | -0.1002832 | | | 0.1361084 | 0.0490056 |
| **PCS(R)** | 0.1151239 | | 0.0055719 | **0.8425709** | | | 0.0324509 | 0.0269198 |
| **PCS(M)** | 0.0182493 | | 0.0609084 | **0.9334730** | | | 0.0334168 | -0.0905804 |
| **PCS(H)** | -0.0604199 | | 0.0307351 | **0.8566090** | | | 0.0265412 | 0.1380779 |
| **SF36(PF)** | **0.6733634** | | 0.1644234 | 0.0196261 | | | 0.1004566 | -0.4028870 |
| **SF36(PH)** | **0.7094073** | | 0.1186078 | -0.0392796 | | | 0.0892508 | -0.0624895 |
| **SF36(EP)** | **0.7884761** | | 0.0366737 | 0.0977453 | | | -0.1750177 | -0.0207307 |
| **SF36(EF)** | **0.7753548** | | -0.0168216 | -0.0386067 | | | 0.1019787 | 0.1113146 |
| **SF36(E)** | **0.7992535** | | -0.1564045 | 0.1015777 | | | -0.1445349 | 0.2852896 |
| **SF36(SF)** | **0.7536360** | | 0.0028377 | 0.0181402 | | | -0.0697790 | 0.0637001 |
| **SF36(GH)** | **0.5381934** | | -0.0661540 | -0.0300044 | | | -0.0018053 | 0.2523995 |
| **TUG** | -0.0508260 | | -0.1175723 | 0.1034888 | | | **0.9309554** | -0.0030743 |
| **6MWT** | 0.0403210 | | -0.0500505 | 0.0346415 | | | **-0.6220702** | -0.1355920 |
