# Supplementary material for: Prognostics for pain in osteoarthritis: Do clinical measures predict pain after total joint replacement?
Source: PLoS One. 2020 Jan 8;15(1):e0222370. doi: 10.1371/journal.pone.0222370 (PMC6948829; doi:10.1371/journal.pone.0222370)
Supplement: S2 Table — A composite measure of pain intensity was built averaging the four outcome scales. Prediction models of our aggregate pain intensity measure show differences across absolute and relative measures (% residual pain). Physical performance was a common predictive factor of both measures. No large effect size was captured in these models, rendering the 4 measures together are not capturing important predictive information. b, unstandardized regression coefficient; SE standard error; β, standardized regression coefficient; F, obtain F-value; t, obtained t-value; R2, proportion variance explained. All statistics are from the final step of the model. **p ≤ 0.01. As the pre-surgical parameters predicting post-surgical pain or residual pain for four pain outcome measures captured distinct independent variables, we reasoned that each may be reflecting specific characteristics and thus combining all four measures would predict larger variance and incorporate the component characteristics. Therefore, we constructed the composite, average score, of all four pain outcome measures and studied its properties. We tested how pre-surgical factors predict 6-months post-surgical KOA pain, using our aggregate measure (S2 Table), again modeling pain and residual pain for KOA patients. For post-surgical aggregated pain severity, the model explained 19% of the variance and included worse health state, lower degree of structural articular damage, and poor results in the physical performance tests. For residual pain, the model explained 7% of the variance and Physical Performance was the only predictive factor. Using these variables to predict HOA post-surgical pain and residual pain we could not find any statistically significant models. (DOCX) [file pone.0222370.s004.docx]

| **Model** | **b** | **SE** | **β** | **t** | **p** | **Adjusted R^2^** |
| --- | --- | --- | --- | --- | --- | --- |
| **Post-surgical Pain** | | | | | | |
| Health | .374 | .161 | .241 | 2.328 | .022 |  |
| Kellgreen-Lawrence Scale | -.408 | .169 | -.213 | -2.416 | .017 |  |
| Physical Performance | .365 | .176 | .215 | 2.067 | .041 |  |
|  |  |  |  |  |  | .192**  F (3,81) = 9.313 |
| **% Residual Pain** | | | | | | |
| Physical Performance | 7.457 | 2.708 | .291 | 2.754 | .007 |  |
|  |  |  |  |  |  | .073**  F (3,81) = 7.584 |
